# 

**Published:** 1919-05

**Authors:** 


[illegible] ENCEPHALITIS

### Texas Medical Journal

ARMY
MEDICAL
SEP 2 3 1947
LIBRARY

SUMMER VACATIONS, HEALTH RESORTS AND SANITARIUMS

Entered at the postoffice at Austin, Texas, as second-class mail matter

